# Reply: “Letter to the Editor Re: Oh J., et al. *Nutrients* 2019, *11*, 343”

**DOI:** 10.3390/nu11030668

**Published:** 2019-03-20

**Authors:** Jongwon Oh, Hyung-Doo Park, Su-Young Kim, Won-Jung Koh, Soo-Youn Lee

**Affiliations:** 1 Department of Laboratory Medicine and Genetics, Samsung Medical Center, Sungkyunkwan University School of Medicine, 81 Irwon-ro, Gangnam-gu, Seoul 06351, Korea; tltc@naver.com (J.O.); nayadoo@hanmail.net (H.-D.P.); 2 Division of Pulmonary and Critical Care Medicine, Department of Medicine, Samsung Medical Center, Sungkyunkwan University School of Medicine, 81 Irwon-ro, Gangnam-gu, Seoul 06351, Korea; suyoung5505@hanmail.net; 3 Department of Clinical Pharmacology & Therapeutics, Samsung Medical Center, Seoul 06351, Korea

We appreciate Hernández-Garduño’s interest in our recent research article [1]. Hernández-Garduño noted the lack of controls with vitamin deficiencies (except for vitamin D) and the small number of underweight controls (*n* = 4) in our study and wondered how the sample size was calculated. Previous studies conducted on Koreans have shown fewer vitamin E or B_12_ deficiencies in healthy controls [2,3], and it was difficult to recruit sufficient vitamin deficienct groups. Regarding the small number of underweight controls, nontuberculous mycobacteria pulmonary disease (NTM-PD) is more common in slender women and healthy subjects are generally not underweight [4]. Regarding the sample size, our research was the first comprehensive comparative investigation of vitamin status in patients with NTM-PD, compared with age- and sex-matched healthy controls. It was difficult to estimate the appropriate sample size since no previous pilot study had been conducted on this research question. Moreover, our study design had characteristics of both cross-sectional and case-control studies. Patients with NTM-PD were recruited for three years and we calculated the study power retrospectively to determine if our findings were powerful enough for the chosen sample size. The result of the power analysis was >99%, indicating that the chosen sample size (150 cases and 150 controls) was sufficient.

Regarding the odds ratios, we examined factors associated with vitamin concentrations, as shown below (Supplementary Table 1). The odds ratios in Table 2 were obtained after adjusting for confounding variables (BMI, total protein, albumin, total cholesterol, and ALT for vitamin A; age for vitamin D; age, sex, and total cholesterol for vitamin E; BMI for methylmalonic acid; and age, sex, CRP, and albumin for homocysteine).

As suggested, we conducted a multivariable analysis in people with a BMI ≥ 18.5. The odds ratio for vitamin A in people with a BMI ≥ 18.5 was 0.116 (95% CI 0.056–0.240; *p* < 0.001), similar to that of the entire study population. In addition, we performed a statistical analysis of vitamin A concentrations after excluding subjects with a low BMI; the results were not significantly different.

Regarding the analysis by NTM species, this was not the primary objective of our study. Since we focused on assessment of vitamin status in patients with NTM-PD, we excluded analysis by NTM species. However, the median serum vitamin A concentrations by NTM infection etiology were statistically different (1.7 for *M.avium*, 1.5 for *M. intracellulare*, 1.3 for *M. abscessus*, and 1.6 for *M. massiliense*; *p* = 0.010 by the Kruskal-Wallis rank sum test).

Finally, although no variables were statistically related to treatment outcome in the univariable analysis, we conducted an additional multivariable analysis to investigate potential independent factors associated with treatment outcomes; variables with a *p*-value of less than 0.2 in the univariable analysis included vitamin A, vitamin E, homocysteine, ALT, and total protein. The multivariable analysis revealed that total protein was associated with treatment outcomes (adjusted OR 3.15, 95% CI 1.16–8.55; *p* = 0.024).

We hope this letter clarifies the points raised by Hernández-Garduño. We appreciate the opportunity to reply to these important questions.

## Figures and Tables

**Supplementary Table 1:** Factors associated with vitamin concentrations: *p*-values for multivariable analysis.

Parameter	Vitamin A	Vitamin D	Vitamin E	Methylmalonic Acid	Homocysteine
Age	0.119	0.005 *	<0.001 *	0.238	0.001 *
Sex	0.117	0.691	0.012 *	0.809	<0.001 *
BMI	<0.001 *	0.157	0.073	0.001 *	0.520
CRP	0.214	0.665	0.983	0.185	0.018 *
Total protein	<0.001 *	0.308	0.957	0.823	0.051
Albumin	<0.001 *	0.223	0.391	0.550	0.023 *
Total Cholesterol	<0.001 *	0.642	<0.001 *	0.261	0.923
AST	0.164	0.671	0.180	0.705	0.411
ALT	0.006 *	0.809	0.240	0.732	0.923

* *p*-value < 0.05.
